# Association of intimate partner violence and other risk factors with HIV infection among married women in India: evidence from National Family Health Survey 2015–16

**DOI:** 10.1186/s12889-021-12100-0

**Published:** 2021-11-17

**Authors:** Neha Shri, T. Muhammad

**Affiliations:** International Institute for Population Sciences, Mumbai, Maharashtra India 400088

**Keywords:** HIV, Intimate partner violence, Married women

## Abstract

**Background:**

Human immunodeficiency virus (HIV) infection remains an important public health concern in many countries. It is fuelled by gender inequality and disparity, which has resulted in a fundamental violation of women’s human rights. This study aims to find out the association of intimate partner violence (IPV) and other risk factors with the prevalence of HIV infection among married women in India.

**Methods:**

This study is based on data from the India National Family Health Survey (2015–16). Bivariate analysis has been performed to estimate the prevalence of HIV. Logistic regression analysis is conducted to find out the association between IPV, factors such as having alcoholic husband and lifetime partner, and HIV infection among currently married women.

**Results:**

Married women who had faced physical, sexual, and emotional violence from their husbands/partners were almost twice more likely to have tested HIV positive compared to married women who did not face violence [OR: 2.14, CI: 1.08–4.50]. The odds of testing for HIV positive was significantly higher among the married women experiencing IPV and having alcoholic husband [OR: 4.48, CI: 1.87–10.70] than those who did not experience IPV and had non-alcoholic husband. The use of condom did not show any significant association with HIV infection. Again, having more than one lifetime partner had a positive association with HIV infection compared to those with one partner [OR: 2.45, CI: 1.21–4.16].

**Conclusions:**

The study revealed that factors such as experiencing all types of IPV, having an alcoholic husband, increased number of lifetime partners, being sexually inactive, belonging to vulnerable social groups, and urban place of residence are important risk factors of HIV infection among married women in India. The results also suggest that gender-based violence and an alcoholic husband may represent a significant factor of HIV infection among married women and interventions should on focus such vulnerable populations.

## Background

Despite an increase in the number of people accessing antiretroviral therapy (ART) and a substantial decline in the new HIV (Human Immunodeficiency Virus) infections worldwide [1, 2], HIV continues to be a major public health issue with some regions of the world being heavily affected [3]. The epidemic has been fuelled by gender inequality and disparity, which resulted in the violation of women’s reproductive rights [4, 5]. Recent evidence on the epidemiology of the disease shows that HIV is no longer restricted to the commonly classified ‘most-at-risk population’ or ‘high-risk group, but is also found in the general population [6–8].

A multitude of factors increases women’s vulnerability to HIV acquisition, including biological, behavioral, socio-demographic, cultural, regional, and structural risks [9, 10]. The HIV in India is described as a series of epidemics, widely varied in terms of prevalence levels, risk factors, and transmission patterns [11]. Further, multiple studies highlight that in Indian context, abusive husbands heighten the risk of HIV infection and there is an increased chance of transmitting HIV in the presence of IPV [12]. On the other hand, mounting evidence highlights the relevance of intimate partner violence (IPV) in understanding HIV infection patterns especially among women, both in the South Asian context and elsewhere [13–15]. Studies also have reinforced that women who experience violence face a choice disability in terms of demanding contraceptive use from their partner and engage in riskier sexual behaviour [16]. A number of studies report that abused women have limited ability to negotiate condom use or refuse sex, and cultural norms dictating low levels of sexual communication between spouses and lack of awareness further worsen this [12, 17, 18].

A diverse body of literature has explored the association between intimate partner violence, with various degrees of emphasis on causal pathways, and have suggested a positive association between women’s experience of intimate partner violence and the risk of HIV. Studies based on women who are present at health clinics often have reported a significantly higher prevalence of violence among HIV-positive women compared with HIV-negative women [19, 20]. Also, women who experience IPV have higher odds of depression, anxiety, and other mental health disorders as well as sexually transmitted infections including HIV [21–23]. The clinical studies have also found women’s HIV status to be associated with their experience of physical violence, physical or sexual violence, and any intimate partner violence [13] and spousal control [24].

A growing body of literature suggests that men who perpetrate violence show higher rates of risky sexual behaviors such as no or inconsistent condom use, having extramarital and multiple sex partners, and forced unprotected sex and they are more prone to sexually transmitted infections (STIs) [25, 26]. A couple of studies have conducted an objective assessment of HIV infection in examining associations of IPV within voluntary counselling and STI clinics with women’s HIV infection and have revealed that HIV infection among women was elevated by experiencing violence from their partners [27, 28]. Similarly, some qualitative studies have illustrated how an inability to negotiate for a safer sex in HIV-negative individuals result in their increased risk of HIV infection by experiencing IPV [29–32].

Chin (2013) in his study using a set of nationally representative data in sub- Saharan Africa shows how the risk of domestic violence is affected by exogeneous increase in HIV prevalence among women in a cluster. He found that the epidemic can affect the risk of partner violence as a direct stressor. For example, HIV prevalence may increase the risk of partner violence by differential levels of conflicts over condom use [33]. Studies also suggest that women find it difficult to negotiate condom use with their life-time partners who consider the request for using it from their wives as an accusation of their infidelity or a claim of autonomy from the woman herself, which may provoke violence [34, 35]. However, another study suggests that women’s HIV infection was not related to their sexual behavior based on a report of these behaviors as in the Indian HIV epidemic among married women is mostly driven by men’s behaviour [36]. There is also evidence that shows the associations among alcohol, violence, and risky sexual behaviors such as forced sex and multiple sexual partners [37].

Despite a great deal of previous research on the impact of spousal violence on HIV infection, several limitations stand out. The majority of studies are based on either the HIV-infected individuals or those who are at an increased risk for HIV infection, considering many of the studies in low-income countries which are conducted in different clinical settings or used samples of highly vulnerable populations [38–41]. However, it should be noted that IPV might not be confined to those who are at greater risk of getting infected, but the link could also be functioning among general population. This study overcomes these shortcomings by identifying the relationships between violence on the risk of HIV prevalence, using large nationally representative data. Although a similar study by Silverman and colleagues has explored the relationship of women’s experience of IPV and HIV infection, this study takes into account the husband’s alcohol use and its interaction with IPV [13]. Despite a substantial progress has been made in terms of narrowing gender gap in the society and improved access to ART, it is necessary to understand the factors that make women more vulnerable to contract HIV infection. This study aims to advance the pre-existing knowledge of HIV prevalence and attempts to find the association between lifetime IPV and other factors including having an alcoholic husband with the prevalence of HIV infection among married women in India.

## Methods

### Data

This study is based on nationally representative data from the fourth round of India’s National Family Health Survey (NFHS, 2015–2016) conducted by the International Institute for Population Sciences, Mumbai under the stewardship of the Ministry of Health and Family Welfare (MoHFW), Government of India. NFHS provides comprehensive information on several aspects of demographic and health indicators at the national, regional, state, and district levels of India. NFHS-4 covered a nationally representative sample of 601,509 households, 699,686 women aged 15–49 years, and 103,525 men aged 15–54 years in India. It is worthwhile to mention that NFHS-4 has adopted a modular approach where the information on sexual behaviors has been collected from women of age 15–49 and men age 15–54, irrespective of their marital status, only from the households selected for the state module. Further, data was collected through trained research assistants with prior informed consent of participating in a national study. As a privacy protection, field staffs were free to reschedule the interview to another time to carry out the interview in private. To ensure that information ondomestic violence module was collected in a uniform way, only one eligible woman per household was randomly selected in accordance with WHO’s guidelines on the ethical collection. IPV was assessed via self-report according to WHO recommendations. For information on HIV, the protocol for blood specimen collection and analysis was anonymous and the test result was merged in the individual data after removing the information which might identify an individual. Finally, the respondents were provided with information and referrals for availing related services. A detailed description of the process of selecting households and respondents has been presented in the national report of NFHS-4, which has been kept in the public domain (http://rchiips.org/nfhs/NFHS-4Report.shtml) [42].

As the objectives of this study deal with testing positive for HIV, the analysis is based on a sub-sample of currently married women aged 15–49 years. Around 96.6% of the currently married women aged 15–49 years gave their consent for HIV testing and 0.1% refused to provide blood for HIV testing (NFHS, 2015–16). Depending on their consent for HIV testing of the blood sample, and nature of the analytic sample, response rates of HIV testing are not calculated directly from the final sample. A total of 60,657 currently married women were tested for HIV. However, the analytic sample was confined to currently married female participants for whom the HIV test results and information on IPV was available. After removing all the missing values based on completion of IPV survey module and available HIV test results, 58,409 currently married women were found eligible for this study.

### Variable description

#### Outcome variable

The outcome variable for the present study was‘HIV test result’, recoded as 0 “persons with HIV” and 1“persons without HIV”.

#### Independent variables

There were two key independent variables in the study. First, in the survey, every woman who was interviewed for the domestic violence module reported her experience of violence and their response was recoded in three major domains i.e. physical violence, emotional violence, and sexual violence. The respondents were categorized to be experiencing physical violence if their husband did any any of these to them (a) pushed, shaked or thrown something at her (b) twisted her arm or pulled her hair (c) slapped her (d) punched her with his fist or something harmful (d) kicked, dragged or beaten her (e) tried to choke or burn her on purpose (f) threatened or attacked her with a knife, gun or any other weapon. If the women reported positively to any of these indicators(a) being physically forced to have sexual intercourse when she did not wanted to (b) forced to perform any sexual acts when she did not want to” the women was categorized as experiencing sexual violence. The respondent was considered to be experiencing emotional violence if they answered yes to any of these behaviour from their spouse (a) said or did something to humiliate her in front of others (b) threatened to hurt or harm her or someone close to her and (c) insulted her or made her feel bad about herself. In the present study, if a married woman aged 15 to 49 ever experienced all of the physical, emotional, and sexual violence from husband was considered to be experiencing Intimate Partner Violence (IPV) and was recoded as 0 ‘no’ and 1 ‘yes’. Further, the respondents were asked if their husbands drink alcohol and their response was coded as 0 “no” and 1 “yes”.

Other independent variables include, ever use of condom/nirodh, coded as 0 “no” and 1 “yes”, and the number of lifetime sexual partners which was coded as 0 “one” and 1 “more than one partner”. Recent sexual activity was recoded as 0 “active for last 4 weeks”, and 1 “not active”. Also, age was grouped into “15–24”, “25–39”, “30–34”, “35–49”and “40–49” [43], and children alive variable was categorized as “No” if women did not have any child alive, “Single” if women had only one child alive and “More than One” if she had more than one alive child. Educational status was recoded as 0 “No” standing for not having received any formal education, and “primary”, meaning that they had received at least primary level of education, “secondary” and “higher”; wealth index was categorised as “poorest”, “poorer”, “middle”, “rich” and “richest”. Access to electricity and television was recoded as “No” and “Yes”. Castes were recoded into “SC” (scheduled caste), “ST” (scheduled tribe), “OBC” (Other Backward Class) and “Other” which is a category that is identified as having higher social status [44]. Further, religion was recoded into “Hindu”, “Muslims”, “Christian” and all other religions as “Others” . Place of residence was recoded as “rural” and “urban” [42].

#### Statistical approach

Descriptive statistics and bivariate analyses have been performed to estimate the prevalence of HIV and intimate partner violence. Further, multivariate analysis (binary logistic) has been conducted to find out the association of lifetime IPV and other background variables with HIV infection among currently married women in the study. The results are presented in the form of odds ratio (OR) with a 95% confidence interval (CI).

The equation for logistic regression is as follows:

$$ \ln \left(\frac{P_i}{1-{P}_i}\right)={\beta}_0+{\beta}_1{x}_1+\dots +{\beta}_M{x}_{m-1}, $$

Where, *β*_0_, …. . , *β*_*M*_, are regression coefficients indicating the relative effect of a particular explanatory variable on the outcome variable.

The multivariable analysis had two models to explain the adjusted estimates. Model-1 provides the adjusted estimates for the control variables. Model-2 provide the interaction effects [45, 46] for IPV and having alcoholic husbands with HIV infection among married women. An “interaction variable” is a variable constructed from an original set of variables to represent either all of the interaction present or some part of it [45–47].

## Results

**Fig. 1:**
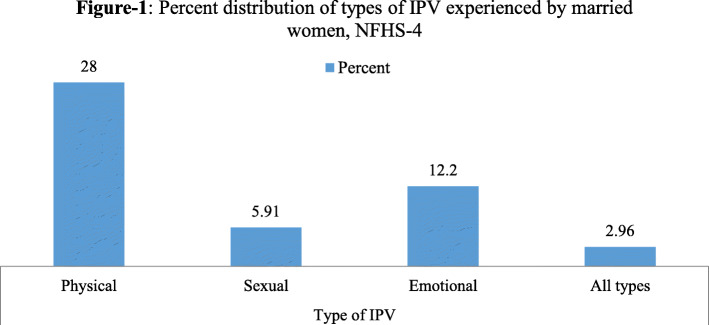
Percent distribution of types of IPV experienced by married women, NFHS-4

Analysis indicates that around 3 % of the married Indian women who were meeting the inclusion criterion of the study were experiencing intimate partner violence (Table 1). Moreover, 0.20% (*n* = 113) currently married women were tested with HIV positive. Around one-third of the eligible women reported that their partners drink alcohol. The vast majority of women participants reported having a single lifetime sexual partner (96.27%) and 85% of the currently married women reported that they have never used condoms/nirodh. Further, three-fourth of the women reported being sexually active in last 4 weeks (72%) and a similar proportion had more than one child who was alive. Figure 1 represents the percent of married women who experienced several types of IPV. It is found that 28% of the study participants had experienced physical violence, whereas, 5.91% of the participants reported sexual violence and 12.2 reported emotional violence. Also, 2.96% had ever experienced all three types of violence from their partners.

**Table 1:** Background characteristics of currently married women who participated in the violence module of the NFHS-4 Women’s Survey (*n* = 58,409) in India

Background variables	Sample	Percentage
HIV		
No	58,296	99.80
Yes	113	0.20
IPV		
No	56,680	97.04
Yes	1729	2.96
Alcoholic husband		
No	40,281	68.96
Yes	18,128	31.04
No. of lifetime partner		
One	56,230	96.27
More than one	2179	3.73
Ever used condom/nirodh		
Yes	8660	14.83
No	49,749	85.17
Sexually active		
Yes	42,172	72.19
No	16,237	27.81
Age group		
15–24	9732	16.66
25–29	12,855	22.01
30–34	12,308	21.07
35–39	10,026	17.17
40–49	13,488	23.09
Number of children alive		
No	5314	9.10
Single	10,991	18.82
More than one	42,104	72.08
Education		
No	19,194	32.86
Primary	8517	14.58
Secondary	25,251	43.23
Higher	5447	9.33
Husband’s education		
No	10,866	18.60
Primary	8781	15.03
Secondary	31,136	53.31
Higher	7626	13.06
Wealth status		
Poorest	11,403	19.52
Poorer	12,417	21.26
Middle	12,183	20.86
Rich	11,577	19.82
Richest	10,829	18.54
Access to electricity		
No	6217	10.92
Yes	50,716	89.08
Access to television		
No	19,565	34.36
Yes	37,368	65.64
Religion		
Hindu	44,423	76.06
Muslim	7712	13.20
Christian	3527	6.04
Others	2747	4.70
Caste		
SC	10,512	18.00
ST	10,029	17.17
OBC	22,986	39.35
Others	14,882	25.48
Place of residence		
Rural	41,565	71.16
Urban	16,844	28.84

Background characteristics of the eligible women who were tested with HIV positive are provided in Table 2. As evident from the data, 0.46% of the women who experienced intimate partner violence were found to be infected with HIV however, among those women who did not experience IPV, only 0.2% were tested with HIV positive. A little less than thrice of the women were living with HIV (0.34) whose partners drank alcohol in comparison to those women whose partners did not drink alcohol (0.13). A higher proportion of women who had more than one sexual partner (0.55) were tested with HIV positive. With the increase in age, more women were found to be infected with HIV with the highest prevalence among women aged 35–39 years The prevalence of HIV was highest among Christian women (0.82%), and a higher percent of women from SC and STs categories were tested with HIV positive (0.21% and 0.36 respectively).

**Table 2:** IPV and other risk factors positive HIV testing among currently married women in India age 15–49

Background variables	n (%)	***p***-values
IPV		
No	105 (0.19)	< 0.001
Yes	8 (0.46)	
Alcoholic husband		
No	52 (0.13)	< 0.001
Yes	61 (0.34)	
No. of lifetime partner		
One	101 (0.18)	< 0.001
More than one	12 (0.55)	
Ever used condom/nirodh		
Yes	13 (0.15)	0.320
No	100 (0.20)	
Sexually active		
Yes	68 (0.16)	0.004
No	45 (0.28)	
Age group		
15–24	13 (0.13)	0.065
25–29	20 (0.16)	
30–34	30 (0.24)	
35–39	28 (0.28)	
40–49	22 (0.16)	
Number of children alive		
No	11 (0.21)	0.479
Single	26 (0.24)	
More than one	76 (0.18)	
Education		
No	30 (0.16)	0.210
Primary	14 (0.16)	
Secondary	60 (0.24)	
Higher	9 (0.17)	
Husband’s education		
No	23 (0.21)	0.658
Primary	13 (0.15)	
Secondary	58 (0.19)	
Higher	17 (0.22)	
Wealth status		
Poorest	16 (0.14)	0.361
Poorer	23 (0.19)	
Middle	24 (0.20)	
Rich	30 (0.26)	
Richest	20 (0.18)	
Access to electricity		
No	3 (0.05)	0.005
Yes	109 (0.21)	
Access to television		
No	31 (0.16)	0.136
Yes	81 (0.22)	
Religion		
Hindu	67 (0.15)	< 0.001
Muslim	9 (0.12)	
Christian	29 (0.82)	
Others	8 (0.29)	
Caste		
SC	22 (0.21)	< 0.001
ST	36 (0.36)	
OBC	30 (0.13)	
Others	25 (0.17)	
Place of residence		
Rural	64 (0.15)	0.001
Urban	49 (0.29)	

Table 3 shows the results of logistic regression analysis of married women having tested positive for HIV. Model − 1 shows the results from the logistic regression after it taking variables religion, caste, lifetime sexual partner, condom use, sexually active status into account. However model-2 included the above mentioned variable with the interaction effect of IPV and husband drinking status. Results show that experiencing Intimate Partner violence is significantly associated with positive HIV status. It revealed that the married women who had faced IPV from their husbands/partners were almost twice more likely to have tested HIV positive compared to married women who did not suffer from violence [OR: 2.14, CI: 1.01–4.5]. The likelihood of testing for HIV positive was significantly higher among the married women whose husbands drink alcohol [OR: 2.33, CI: 1.56–3.47]. Further, prevalence of HIV was 2.25 times higher among women who had more than one lifetime partner in comparison to those who had one partner [OR: 2.25, CI: 1.21–4.16]. Interestingly, the use of condoms did not show any significant association with positive HIV status. Women aged 30–34 years and 35–39 years were twice significantly more likely to be living with HIV than women aged 15–19 years [OR: 2.11, CI: 1.00–4.44; OR: 2.29, CI: 1.07–4.94].

**Table 3:** Logistic regression estimates for testing HIV positive among currently married women in India aged 15–49 years

Background variables	Model 1	Model 2
	AOR (95% CI)	AOR (95% CI)
IPV		
No	Ref.	
Yes	2.140** (1.018–4.500)	
Alcoholic husband		
No	Ref.	
Yes	2.333*** (1.566–3.476)	
No. of lifetime partner		
One	Ref.	Ref.
More than one	2.248** (1.213–4.166)	2.246** (1.212–4.163)
Ever used condom/nirodh		
Yes	Ref.	Ref.
No	1.296 (0.689–2.437)	1.299 (0.691–2.443)
Sexually active		
Yes	Ref.	Ref.
No	1.785*** (1.209–2.635)	1.789*** (1.212–2.642)
Age group		
15–24	Ref.	Ref.
25–29	1.401 (0.654–3.000)	1.400 (0.654–2.999)
30–34	2.111** (1.004–4.441)	2.112** (1.004–4.442)
35–39	2.294** (1.065–4.941)	2.295** (1.066–4.943)
40–49	1.302 (0.583–2.908)	1.300 (0.582–2.904)
Number of children alive		
No	Ref.	Ref.
Single	1.036 (0.492–2.183)	1.038 (0.493–2.187)
More than one	0.700 (0.344–1.423)	0.700 (0.344–1.423)
Education		
No	Ref.	Ref.
Primary	0.984 (0.501–1.933)	0.983 (0.500–1.931)
Secondary	1.204 (0.673–2.156)	1.204 (0.673–2.155)
Higher	0.767 (0.292–2.016)	0.768 (0.292–2.018)
Husband’s education		
No	Ref.	Ref.
Primary	0.524* (0.253–1.086)	0.524* (0.253–1.086)
Secondary	0.671 (0.371–1.213)	0.669 (0.370–1.210)
Higher	0.917 (0.406–2.073)	0.915 (0.405–2.067)
Wealth status		
Poorest	Ref.	Ref.
Poorer	0.998 (0.481–2.069)	1.001 (0.483–2.075)
Middle	1.009 (0.439–2.320)	1.012 (0.440–2.326)
Rich	1.195 (0.483–2.953)	1.198 (0.485–2.959)
Richest	0.918 (0.327–2.573)	0.922 (0.329–2.585)
Access to electricity		
No	Ref.	Ref.
Yes	4.214** (1.238–14.35)	4.200** (1.234–14.30)
Access to television		
No	Ref.	Ref.
Yes	0.893 (0.506–1.577)	0.894 (0.506–1.579)
Religion		
Hindu	Ref.	Ref.
Muslim	0.839 (0.386–1.822)	0.835 (0.384–1.814)
Christian	3.661*** (2.081–6.442)	3.657*** (2.079–6.433)
Others	1.664 (0.775–3.573)	1.665 (0.775–3.573)
Caste		
SC	Ref.	Ref.
ST	0.908 (0.488–1.690)	0.907 (0.487–1.686)
OBC	0.631 (0.356–1.118)	0.630 (0.356–1.117)
Others	0.858 (0.465–1.583)	0.856 (0.464–1.580)
Place of residence		
Rural	Ref.	Ref.
Urban	1.808*** (1.170–2.795)	1.816*** (1.175–2.807)
IPV# Alcoholic husband		
No# No		Ref.
No# Yes		2.408*** (1.604–3.615)
Yes# No		3.615* (0.867–15.07)
Yes# Yes		4.477*** (1.873–10.70)

*** *p* < 0.01, ** *p* < 0.05, * *p* < 0.1; *AOR* Odds Ratio Adjusted for all the covariates in the study, *CI* Confidence Interval, *Ref* Reference category, *SC* Scheduled Caste, *ST* Scheduled Tribe, *OBC* Other Backward Class

Although we did not find any statistical significance, condom or nirodh use by the husband was positively associated with having HIV infection. Respondents who did not report of using condom/nirodh were 1.30 times higher likely to be HIV positive [OR: 1.30, CI: 0.69–2.43]. A strong positive association between sexually inactive in last 4 weeks and having HIV infection was found among married women [OR: 1.79, CI: 1.21–2.64]. women who had access to electricity were 4.14 times more likely to be living with HIV in comparison to those who did not have access to electricity [OR: 4.21, CI: 1.24–14.35].. Urban residents were 1.81 times significantly more likely to have HIV infection than their rural counterparts [OR: 1.81, CI: 1.25–2.85]. Moreover, married women from Christian religion [OR: 3.66, CI: 2.08–6.44] were significantly more likely to be HIV infected compared to the married women from other religion. Model-2 represents the interaction effect of intimate partner violence along with alcohol use by their husband on HIV infection among currently married women. Women women who experienced IPV and had an alcoholic partner were 4.48 times significantly higher likely to have HIV infection than women experiencing IPV and having a alcoholic partner [OR: 4.48, CI: 1.87–10.70].

## Discussion

Findings from the current study show that women who have been facing violence in the form of sexual, physical, and emotional are more likely to be HIV infected than their counterparts. This finding both corroborates and contrasts with prior works from the South African context. However similar finding has been observed from a study [13] evidenced from NHS-3data. A population-based study of married women in Rwanda [48], revealed a significant and strong association between HIV and emotional IPV and sexual IPV, respectively, as well as between HIV infection and a total violence score. Significantly more women who experienced physical or sexual IPV were HIV-positive in a 2007 study of women attending an HIV/STI clinic in Bangalore [28].

Studies using the same data (NFHS, 2005–06), found associations of reporting IPV with the testing positive for HIV only for specific subgroups, for instance, study by Ghosh et al., [12] observed no significant association of sexual violence with HIV infection in ever-married women, but a positive association was found among currently married women. On the other hand, Silverman et al., found that married women who had experienced both sexual and physical forms of IPV had a higher chances of testing positive for HIV compared to those who did not experience any forms of IPV [13]. However, in some studies, no difference in HIV positive status was observed among those who experienced physical without sexual IPV compared with those who experienced no IPV [49, 50]. furthermore, in parallel to current findings, a previous study suggests that people who experience IPV may have increased risk for having tested positive for HIV by voluntarily engaging in risky sexual behaviors that includes having multiple partners, unprotected sex, and using substances [20].

Studies in less-developed countries show that individuals with drinking behaviour may have casual sex without using a condom or any safety and are more likely to engage in transactional sex with multiple sex partners resulting in risk for HIV [38, 51, 52]. Consistently, the results showed that married women whose husbands drink alcohol were at greater risk for testing positive for HIV infection. Besides, married women in an Indian study who faced alcohol-related violence from their husbands were also more prone to have more sexual risks including HIV [37]. The same study also suggests that women in such a country where divorce is not considered as a viable option are often forced to have sex and such behaviors are endorsed in the society due to prevailing gender norms. Furthermore, the interaction effect of IPV and having an alcoholic husband on being tested HIV positive in the present study showed that married women who experienced all types of violence and had an alcoholic husband had significantly higher chances of being infected with HIV compared to their counterparts.

Self-reported lifetime number of sexual partners and lifetime history of condom use for contraceptive purposes were considered as sexual risk covariates in past studies. Gupta et al. [53] revealed that the proportion of extramarital affairs among married males (11%) is about five times higher than among females (2%). Given the possibility that husbands contract HIV from extramarital affairs, married women may contract the virus through sexual intercourse within marriage [54]. Similarly, we found that currently, married women who have multiple partners were at increased risk of contracting HIV infection. Although the association was not significant, a positive direction shows that the use of a condom or nirodh, common contraceptives in India [39], was found to be a protective factor against HIV infection. In patriarchal societies, suggesting condom use or refusing unprotected sex in a marriage or cohabiting relationship is seen as questioning male authority [55]. The risk of HIV in marriage and in stable or cohabiting relationships is due to an increased frequency of sex and low condom use [56].

Although education and wealth status showed no significant association with HIV infection in the logistic regression model, bivariate results showed that having education and being from a rich background was not protective against testing positive for HIV among married women in our study. The finding is consistent with previous studies in less-developed countries that found an increased likelihood of HIV infection among women who are educated and have better economic status [57]. Moreover, contrary to previous studies that have shown that access to electricity may increase the awareness and decrease the chances of IPV as well as HIV infection [19, 58], current results show a positive association of access to electricity with HIV infection among married women. The finding needs to be further investigated.

The study has several limitations. Firstly, given that the study is based on a cross-sectional survey, interpretations of our findings are limited to statistical associations. Secondly, the relationships among alcohol drinking, sexual behavior, and violence that may further place women at risk of HIV infection are not taken into account in the analysis. While the study focused on partner violence within marriage, the violence in other relationships, such as premarital, extra-marital, and commercial ones, were not represented. Nevertheless, this study presents rich representative data on a sensitive topic assessed at a national level.

## Conclusion

The findings of the study have shown that factors such as experiencing all types of IPV, having an alcoholic husband, increased number of lifetime sexual partners, being sexually inactive, belonging to vulnerable social groups, and urban place of residence are important risk factors of HIV infection among married women in India. The results also suggest that gender-based violence and an alcoholic husband may represent a significant factor in married women’s vulnerability to acquire HIV in poor resource-settings and interventions should not only encompass the health sector, but also extend beyond that to include more vulnerable populations. Further, longitudinal and qualitative studies are warranted for tracking married women’s sexual negotiation after facing partner violence over time and exploring their lived risky sexual experience within their marriage.

## Data Availability

The study utilises secondary source of data which is freely available in public domain through https://www.dhsprogram.com/

## Abbreviations

IPV: Intimate partner violence
HIV: Human immunodeficiency virus
NFHS: National Family Health Survey
OR: Odds ratio
CI: Confidence interval
